# Claw sign of brachial plexopathy on ^18^F-FDG PET/CT in neurolymphomatosis following successful treatment of lymphoma

**DOI:** 10.1186/s41824-022-00132-7

**Published:** 2022-06-01

**Authors:** Simon Doran, Gerard Lambe, Afshin Nasoodi

**Affiliations:** 1 Radiology Department, St James Hospital, Dublin, Ireland; 2 Department of Radiology, Mater Misericordiae University Hospital, Dublin, Ireland

**Keywords:** PET/CT, Oncology imaging, Neurolymphomatosis, Non-Hodgkins lymphoma, Brachial plexus

## Abstract

Neurolymphomatosis is a rare neurological manifestation associated with non-Hodgkin’s lymphoma. Here we present a case of brachial plexus neurolymphomatosis in a patient with relapsed non-Hodgkin’s lymphoma exquisitely demonstrated on ^18^F-FDG PET/CT. It highlights the characteristic imaging features and importance of multimodality imaging in diagnosing neurolymphomatosis.

Neurolymphomatosis is a rare neurological complication of Non-Hodgkin’s lymphoma (NHL), often clinically presenting as a radiculopathy (Grisariu et al. 2010; Baehring et al. 2003).

We present the imaging findings in a 60-year-old Caucasian man with stage III NHL who underwent routine surveillance with PET using fluorodeoxyglucose (^18^F-FDG) integrated with computed tomography (^18^FDG-PET/CT) following initial complete (Deauville 1) metabolic response to standard chemotherapy regimen.

^18^F-FDG-PET/CT demonstrated claw-shaped asymmetrical right sided, multi-segmental intense linear ^18^F-FDG uptake, in the distribution of C5 to T1 nerve roots with extension of abnormal uptake along the trunks and cords of the right brachial plexus, most elegantly displayed on the maximum intensity projection (MIP) image (Fig. 1, thick arrow) and confirmed on the multiplanar fused images (Figs. 2, 3). No structural abnormality was evident on the CT component of the study. New nodal disease was illustrated in the upper abdomen (Fig. 1, curved arrow) without central nervous system (CNS) involvement. At the time of ^18^F-FDG PET/CT imaging, the patient had right upper limb weakness and neuropathic pain which had been ongoing for 1 month, although this vital clinical information had not been disclosed on the referral letter. Of note, an MRI with dedicated brachial plexus protocol, performed 3 weeks earlier, had failed to identify any abnormality. In the context of previously treated NHL and scintigraphic evidence of right-sided radiculopathy on ^18^F-FDG PET/CT, a diagnosis of neurolymphomatosis was made. This diagnosis was subsequently endorsed following discussion with the referring clinician and disclosure of the relevant clinical information.

**Fig. 1:**
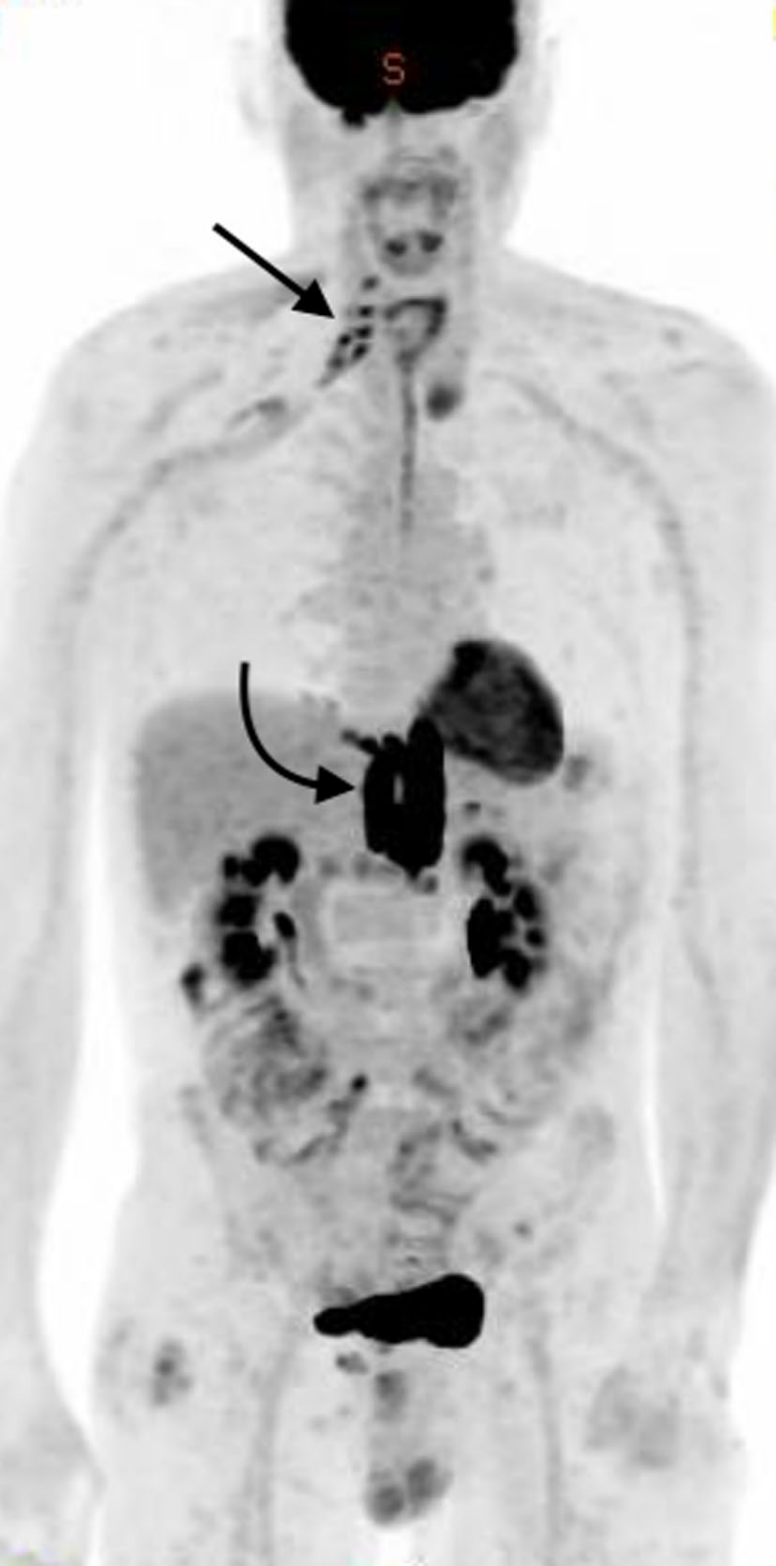
MIP image from whole-body ^18^F-FDG-PET/CT demonstrates claw-shaped asymmetrical right sided, multi-segmental intense linear ^18^F-FDG uptake, in the distribution of C5 to T1 nerve roots of the brachial plexus (straight arrow) and nodal recurrence below the diaphragm (curved arrow)

**Fig. 2:**
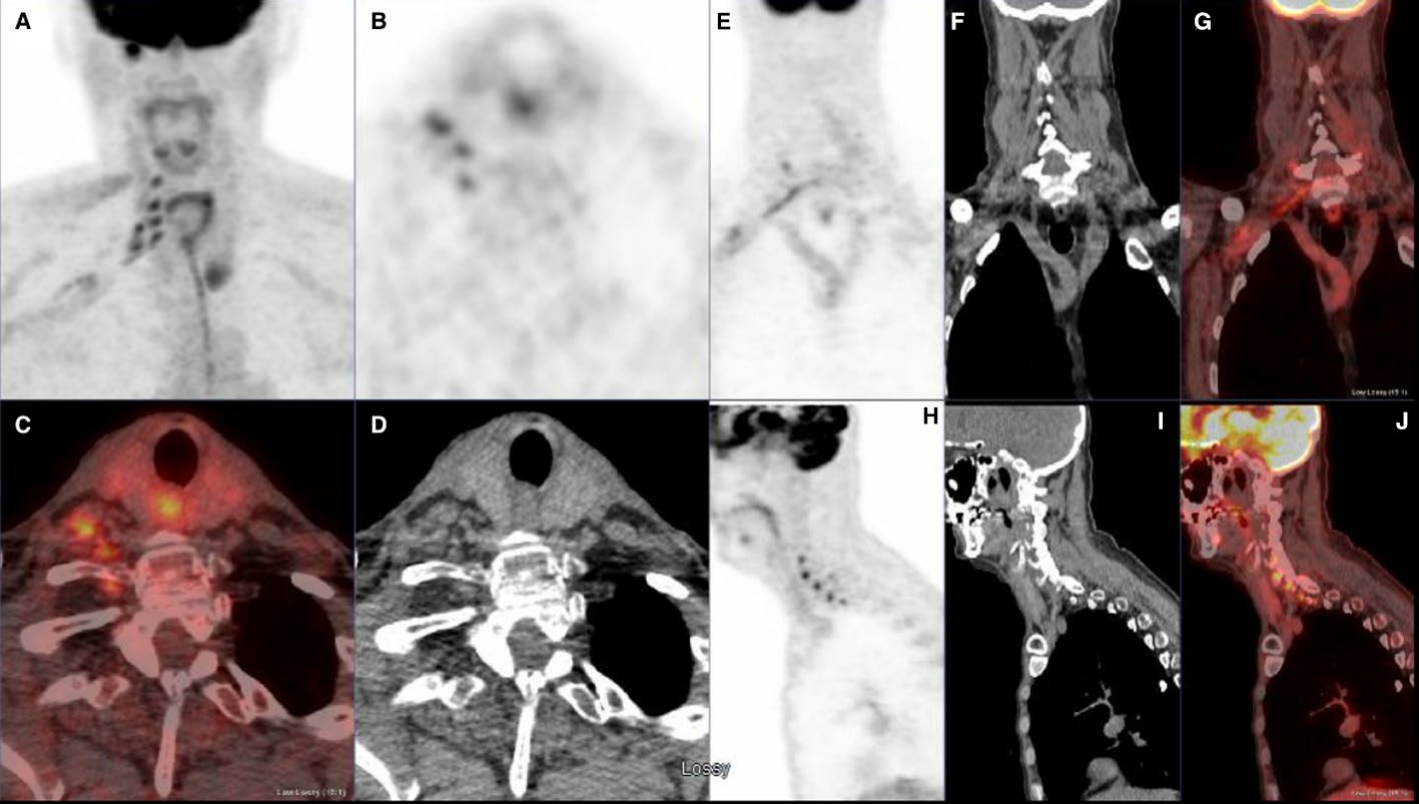
Axial PET, fused and CT images (**B**, **C** and **D**) at the level of exit foramina in lower neck. Note intense uptake by the nerve roots on the right from C5 to T1. Coronal PET, CT and fused images (**E**, **F** and **G**) and Sagittal PET, CT and fused images (**H**, **I** and **J**) confirm the uptake in the distribution of the brachial plexus

**Fig. 3:**
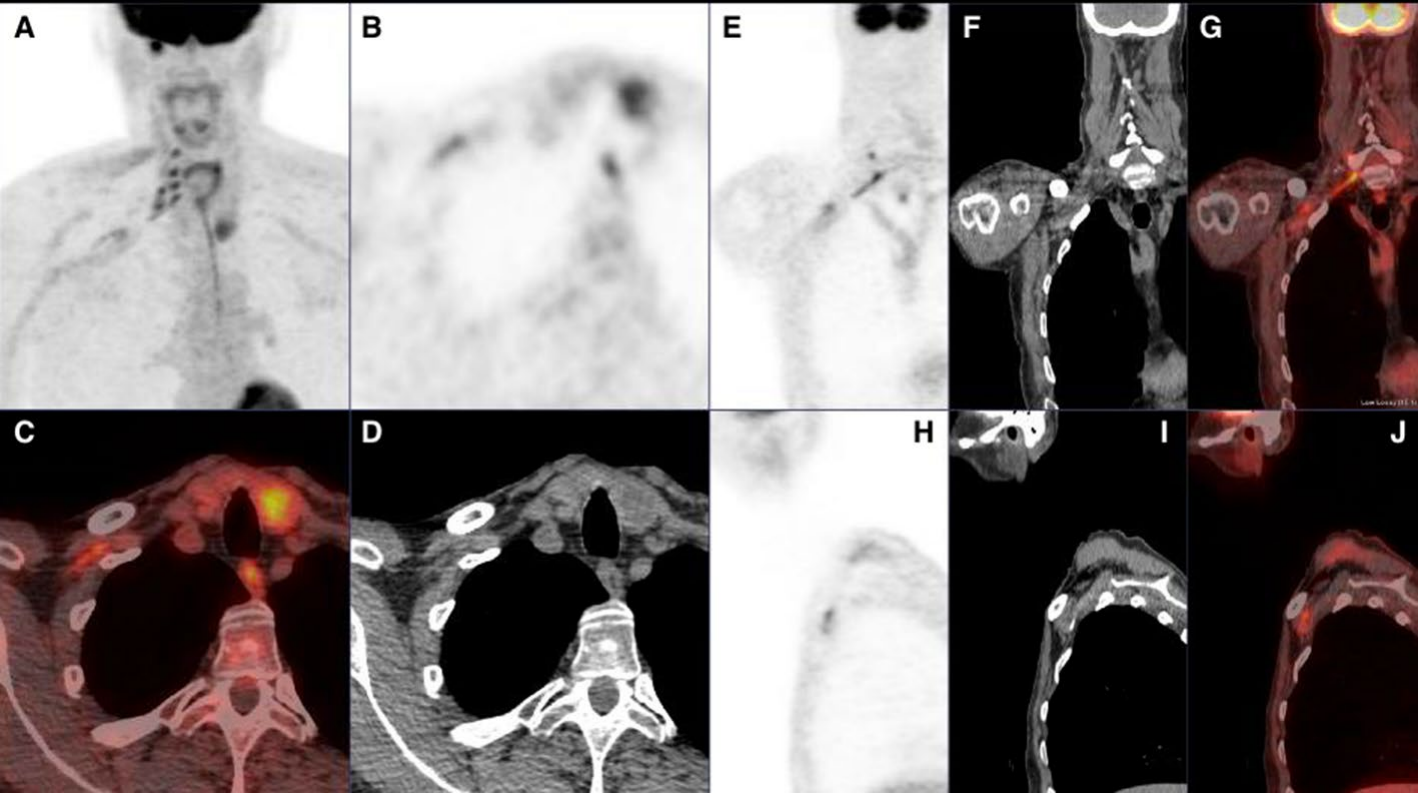
Axial PET, fused and CT images (**B**, **C** and **D**) at the level of the right clavicle. Note intense uptake by the trunks and cords. Coronal PET, CT and fused images (**E**, **F** and **G**) and Sagittal PET, CT and fused images (**H**, **I** and **J**) confirm the uptake in the distribution of the brachial plexus

MRI and ^18^F-FDG PET/CT demonstrate the abnormality in 80 and 88% of cases, respectively (Grisariu et al. 2010). An infiltrative pattern of ^18^F-FDG PET/CT uptake, involving a neural plexus or along a peripheral nerve, is the typical finding at ^18^F-FDG PET/CT (Strobel et al. 1244; Ozturk et al. 2006; Zhou et al. 2014). Accompanying CT images will often be normal; however, occasionally nodular thickening of the affected nerve segment can be identified (Strobel et al. 1244).

^18^F-FDG PET/CT is an essential part of staging and monitoring treatment response in patients with NHL (Johnson et al. 2015). It is exquisitely sensitive to the presence of high-grade lymphoma due to the intense inherent metabolic activity associated with such tumours and is particularly useful for identifying and staging extra-nodal disease (Zhou et al. 2014; Johnson et al. 2015). Neurolymphomatosis is at its essence, extra-nodal lymphomatous infiltration of neural tissue, which in conjunction with the typically small patchy nature of the lesions (Zhou et al. 2014) could potentially explain the diagnostic advantage of ^18^F-FDG PET/CT over MRI. Also relevant is the timeline between the two studies in our case, with MRI performed 3 weeks earlier, arguably too soon to identify the abnormalities seen on the subsequent ^18^F-FDG PET/CT.

Neurolymphomatosis can be treated by a combination of systemic chemotherapy, intrathecal chemotherapy and local radiotherapy (Grisariu et al. 2010). Treatment response is highly variable with a poor median survival following diagnosis of just 10 months (Grisariu et al. 2010; Matsuoka-Kamiya et al. 2014).

MRI is the modality of choice during the initial work-up in patients with peripheral nerve symptoms such as radiculopathy. Novel MR techniques such as whole body diffusion weighted imaging might be helpful to extract the functional information needed to make the correct diagnosis and avoid false negative results (Tanaka et al. 2013). Our case underscores the importance of multi-modality/metabolic imaging in the context of known NHL and persistent symptoms, where there is a strong clinical suspicion of neurolymphomatosis. It is imperative to communicate essential clinical information with the nuclear medicine department to avoid diagnostic ambiguity or mis-interpretation.

## Conclusion

This case highlights the importance of multimodality imaging where there is strong clinical suspicion of neurolymphomatosis. It also demonstrates the characteristic imaging features on ^18^F-FDG PET/CT of brachial plexus neurolymphomatosis. Providing the diagnosticians with contemporary and relevant clinical information is paramount to avoid diagnostic ambiguity and erroneously assigning the scintigraphic findings to a wrong pathological process. MRI remains the diagnostic modality of choice; however, a negative study should not result in loss of confidence of the reporter in interpretation of the abnormalities seen on the ^18^F-FDG PET/CT.

## Data Availability

Not applicable.

## Abbreviations

NHL: Non-Hodgkin’s lymphoma
MIP: Maximum intensity projection
CNS: Central nervous system
